# “I WOULD LIKE TO BE IN CONTROL”: PERSPECTIVES OF OLDER ADULTS ON AUTONOMOUS DRIVING

**DOI:** 10.1093/geroni/igae098.1314

**Published:** 2024-12-31

**Authors:** Sajay Arthanat

**Affiliations:** University of New Hampshire, Durham, New Hampshire, United States

## Abstract

Autonomous driving technology is projected to have major implications on transportation safety, independence and aging-in-place of older adults. However, are older adults ready to embrace driving automations? This mixed-method research study explored the perspectives and readiness of older adults for autonomous driving, and underscored the demographic and age-related factors that potentially influence the adoption. The study involved two aims and employed the Levels of Autonomous Driving developed by the Society of Automotive Engineers to gauge readiness for the technology. In Aim 1, semi-structured interviews were conducted with nine older adults (purposively sampled) following a tutorial depicting the autonomous driving levels. The interviews were guided by the Unified Theory of Acceptance and Use of Technology (UTAUT) primarily focusing on the affinity of the participants to each automation level. A survey was also pilot tested with the participants using cognitive interviewing. In Aim 2, the survey was completed by 528 older adults (65+). The survey uniquely presented 16 realistic driving scenarios with respondents choosing the level of automation preferred for their car in each. Thematic analysis of the qualitative data underscored the perceived benefits, facilitators, barriers and mistrust associated with driving automation. The readiness for automation was generally low in the survey sample and ranged from no automation to driver assistance (52.7%), partial to conditional (35.9%) and high to full automation (11.3%). Pearson correlations showed that readiness significantly varied by income, living status, geographical location and perceived usefulness for aging-in-place. Multivariate analyses is underway to explore causal pathways in the study data.

